# Clinical responses observed with imatinib or sorafenib in melanoma patients expressing mutations in *KIT*

**DOI:** 10.1038/sj.bjc.6605635

**Published:** 2010-04-06

**Authors:** D Handolias, A L Hamilton, R Salemi, A Tan, K Moodie, L Kerr, A Dobrovic, G A McArthur

**Affiliations:** 1 Research Division, Peter MacCallum Cancer Centre, St Andrews Place, East Melbourne, Victoria 3002, Australia; 2 Department of Medicine, St Vincent's Hospital, The University of Melbourne, Melbourne, Victoria 3010, Australia; 3 Department of Haematology, Royal Prince Alfred Hospital, 50 Missenden Road, Camperdown, New South Wales 2050, Australia; 4 Department of Medical Oncology, Sydney Cancer Centre, Royal Prince Alfred Hospital, 50 Missenden Road, Camperdown, New South Wales 2050, Australia; 5 Sydney Melanoma Unit, University of Sydney, Sydney, New South Wales 2006, Australia; 6 Molecular Developmental Research and Development Laboratory, Department of Pathology, Peter MacCallum Cancer Centre, St Andrews Place, East Melbourne, Victoria 3002, Australia; 7 Department of Diagnostic Imaging, Peter MacCallum Cancer Centre, St Andrews Place, East Melbourne, Victoria 3002, Australia; 8 Department of Pathology, The University of Melbourne, Parkville, Victoria 3010, Australia

**Keywords:** melanoma, *KIT*, mutation, imatinib, sorafenib, brain

## Abstract

**Background:**

Mutations in *KIT* are more frequent in specific melanoma subtypes, and response to KIT inhibition is likely to depend on the identified mutation.

**Methods:**

A total of 32 patients with metastatic acral or mucosal melanoma were screened for mutations in *KIT* exons 11, 13 and 17.

**Results:**

*KIT* mutations were found in 38% of mucosal and in 6% of acral melanomas. Three patients were treated with imatinib and one with sorafenib. All four patients responded to treatment, but three have since progressed within the brain.

**Conclusion:**

The observed clinical responses support further investigation of KIT inhibitors in metastatic melanoma, selected according to *KIT* mutation status.

*KIT* has been identified as a of biological importance in melanoma, and mutations (and/or amplification) appear to be largely confined to acral and mucosal subtypes and those associated with chronic sun damage (Curtin *et al*, 2006; Beadling *et al*, 2008; Satzger *et al*, 2008). Clinical experience in patients with gastrointestinal stromal tumour (GIST) indicates that sensitivity and resistance patterns to the KIT kinase inhibitors can be predicted from the presence and location of specific *KIT* mutations (Heinrich *et al*, 2006). Patients with melanomas containing activating mutations in *KIT* have been reported to respond to imatinib therapy (Lutzky *et al*, 2008; Quintas-Cardama *et al*, 2008; Satzger *et al*, 2010); however, it remains uncertain whether mutations considered to be predictive of imatinib resistance can respond to second-generation KIT kinase inhibitors.

This report describes the frequency of *KIT* mutations in a prospectively selected group of Australian melanoma patients identified as ‘at risk’ of harbouring a *KIT* mutation based on clinical subtype. Four case reports illustrating significant clinical responses to kinase inhibitors highlight the therapeutic potential of *KIT* mutation screening in advanced melanoma. However, central nervous system (CNS) progression following systemic responses in three patients raises an important issue pertaining to treatment efficacy in the setting of brain metastases.

## Materials and methods

### Patient population

Patients from two melanoma centres in Australia (Peter MacCallum Cancer Centre, Melbourne and Royal Prince Alfred Hospital, Sydney) provided informed consent for *KIT* mutation screening if they had history of primary acral or mucosal melanoma between October 2006 and December 2008. Patients with metastatic melanoma requiring treatment were considered for a phase II clinical trial of imatinib (http://www.clinicaltrials.gov; NCT identifier: 00171912). Eligibility included the identification of an activating mutation in *KIT* predicted to be sensitive to imatinib, measurable disease as assessed by RECIST (Response Evaluation Criteria in Solid Tumours), normal organ function and an ECOG performance status between 0 and 2. Dosing commenced at 400 or 600 mg daily with an option to escalate to 600 or 800 mg depending on the response. Each participating institutional human ethics committee had previously reviewed and approved the study.

### Genotyping

Genomic DNA obtained from 32 melanoma samples (23 metastases and 9 primary tumours) were tested for mutations in *KIT* (exons 11, 13 and 17) using high-resolution melting-screen analysis and confirmed by direct sequencing. Details of methods have been published previously (Handolias *et al*, 2010).

## Results

A total of 32 patient samples (16 mucosal and 16 acral) were analysed for mutations in *KIT* and 7 mutations were detected (Table 1). One acral melanoma contained an exon 17 (D820Y) mutation, representing a mutation frequency of 6% within this subtype. Six mucosal tumours (38%) harboured a *KIT* mutation, and these spanned across all exons tested. Four patients with metastatic mucosal melanoma were treated with a kinase inhibitor, all of whom had heterozygous mutations confirmed by direct sequencing. Three consented to undergo a clinical trial of imatinib, and one patient with an exon 17 (D820Y) kinase domain mutation consented to treatment with sorafenib (off label use), because of predicted resistance to imatinib. All four patients had radiological and/or clinical response to therapy as described in the following case reports and as summarised in Table 2.

### Case report (1)

**Figure 1:**
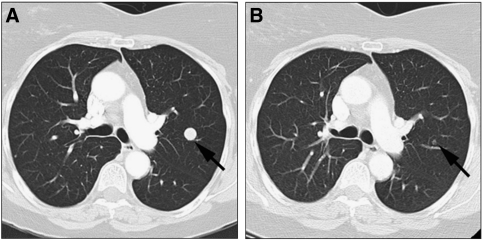
CT chest images of pulmonary metastasis arising from anal melanoma at baseline (**A**) and then at 3 months (**B**) showing reduction in the size of the lesion on imatinib (Case 1).

A 65-year-old woman with a history of anal melanoma and resected splenic and cerebral metastases, developed a new pulmonary lesion 3 years later. A 21 base-pair duplication in exon 11 of *KIT* was identified in the splenic metastasis. On the basis of predicted sensitivity, she was commenced on a 600 mg daily dose of imatinib and a 60% reduction in the pulmonary metastasis was observed at 12 weeks (Figure 1). After a further 3 months of treatment, she underwent whole brain irradiation for intracranial progression. After 1 year of continuing with imatinib, the patient had not developed any new systemic metastases but had advanced further within the brain and died of progressive CNS disease.

### Case report (2)

**Figure 2:**
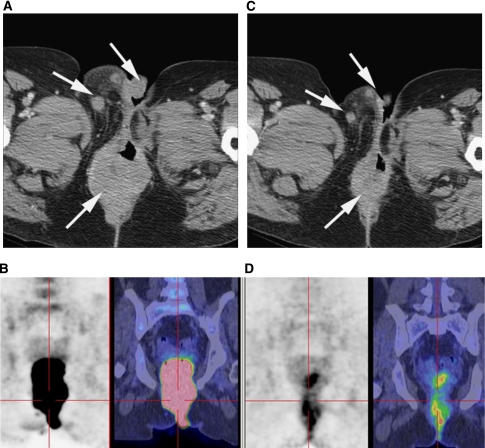
CT pelvis and FDG PET/CT images at baseline (**A**, **B**) and at 1 month (**C**, **D**) after treatment of a metastatic vulval melanoma with imatinib. Arrows indicate areas of response (Case 2).

A 48-year-old woman with a recurrent vulval melanoma containing a K642E mutation in exon 13 of *KIT* was treated with imatinib (400 mg daily dose) following loco-regional relapse 2 years after optimal surgical management and high-dose adjuvant radiotherapy. A significant reduction in the uptake on FDG PET was observed in the local recurrence, and there was complete resolution of soft tissue metastases at multiple sites after 3 weeks (Figure 2). The patient continued to respond to therapy with a 35% reduction in the sum of measured lesions on CT at 12 weeks (image not shown).

### Case report (3)

**Figure 3:**
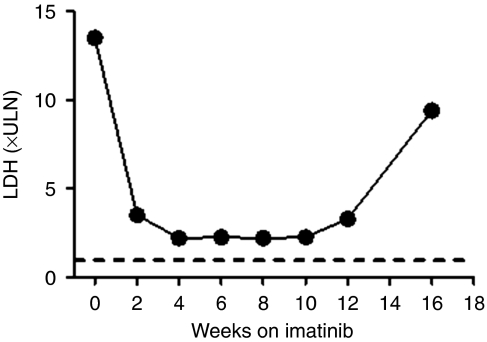
Marked LDH response within 2 weeks of commencing imatinib in a metastatic mucosal melanoma arising from the labia (Case 3). Dashed line indicates upper limit of the reference range.

A 38-year-old woman with a resected labial melanoma had an isolated clitoral recurrence after 3 years. Visceral metastases and a soft tissue thigh mass were diagnosed 2 years later and unresponsive to standard chemotherapy. An L576P mutation in exon 11 of *KIT* was identified in the clitorectomy specimen and imatinib was commenced at a 400 mg daily dose. The patient's symptoms improved dramatically within days and her LDH decreased from 2754 to 276 IU l^−1^ (upper limit of reference range=220 IU l^−1^) (Figure 3). A 42% reduction in the summed size of target lesions was seen on CT (image not shown). The patient then progressed at all sites despite an escalation in the dose of imatinib to 600 mg and ceased treatment after 16 weeks, dying shortly thereafter with new brain metastases.

### Case report (4)

**Figure 4:**
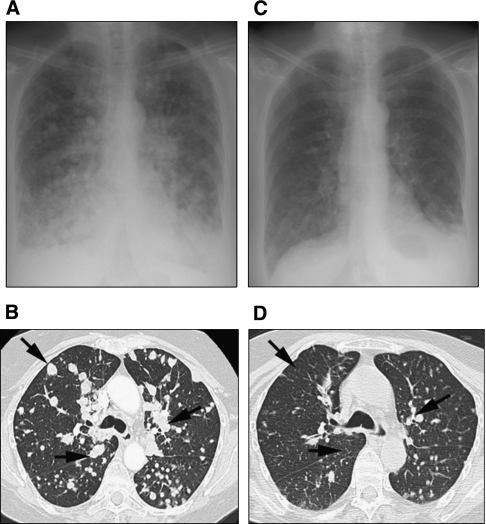
Chest X-ray and chest CT images at baseline (**A**, **B**) and then at 1 month (**C**, **D**) after treatment of a metastatic anal melanoma with sorafenib demonstrates significant reduction in the number and size of pulmonary metastases. Arrows indicate major sites of response (Case 4).

A 62-year-old woman with anal melanoma and widespread pulmonary metastases complicated by impending respiratory failure, had a D820Y mutation in exon 17 of *KIT* in her primary tumour. She did not respond to chemotherapy and was ineligible for imatinib on clinical trial based on predicted resistance. Sorafenib was commenced (400 mg b.i.d.), and within days, the patient was discharged from hospital without supplemental oxygen. Chest imaging at 4 weeks showed a 27% reduction in the sum of target lesions (Figure 4). Treatment was then interrupted because of gastrointestinal toxicity, and within 2 weeks, multiple brain metastases were diagnosed. Despite whole brain radiotherapy and re-introduction of sorafenib, the patient died from progressive CNS disease.

## Discussion

This case series can be seen as proof of two points of principle. First, clinical tumour characteristics have successfully been used to identify a subgroup of melanoma patients with a high prevalence of therapeutically relevant *KIT* mutations. Second, the genetic location of *KIT* mutation has successfully been used to guide selection of the KIT inhibitor in melanoma, echoing the broader experience of the use of *KIT* mutation subtype to select kinase inhibitor therapies in GIST (Heinrich *et al*, 2008).

Tyrosine kinase inhibitors demonstrate variable efficacy against *KIT* mutation variants in GIST. Mutations affecting the juxtamembrane region of the receptor result in its constitutive activation, and are particularly responsive to imatinib, whereas kinase domain mutations often confer resistance (Heinrich *et al*, 2003). The therapeutic responses to imatinib in this series mirror the clinical experience of other melanoma patients with similar sensitising *KIT* mutations (Hodi *et al*, 2008; Lutzky *et al*, 2008). The convincing clinical response observed in the patient with the L576P mutation in exon 11 (Case 3) is interesting to note in the context of *in vitro* data demonstrating reduced sensitivity to imatinib compared with other exon 11 mutations (Antonescu *et al*, 2007). It is possible that in this case, high imatinib concentrations were achievable within the tumour which resulted in the clinical effect. The non-sustainable response to therapy could also be explained by reduced drug levels over time as shown in pharmacokinetic studies of GIST patients wherein imatinib levels decrease with ongoing use because of increased drug clearance (Judson *et al*, 2005). It is likely that in these less-sensitive mutations, increased levels of imatinib or an alternative kinase inhibitor is required. Dasatinib has since been shown to have a selective inhibitory effect in this *KIT* mutation both *in vitro* and in the clinic in the setting of previous imatinib therapy (Woodman *et al*, 2009).

In GIST, mutations in the kinase domain of *KIT* are usually due to secondary point mutations (Kitamura and Hirotab, 2004) and can confer resistance to imatinib due to the altered conformation of the kinase which prevents drug interaction with the ATP binding pocket (Frost *et al*, 2002; Foster *et al*, 2004). The last case report in this series describes the treatment response seen in a patient with a D820Y mutation in exon 17 treated with sorafenib based on *in vitro* data predicting response (Guo *et al*, 2007). Sorafenib inhibits a number of kinases (Wilhelm *et al*, 2004) in addition to KIT, which cannot be excluded as contributing to the response to treatment seen in this patient.

The increased rates of CNS metastases in this series may be explained by the limited penetration of small molecule kinase inhibitors into the brain, as documented in other imatinib-sensitive malignancies such as chronic myeloid leukaemia, Philadelphia chromosome-positive acute lymphocytic leukaemia and GIST in which relapse within the CNS has been reported (Takayama *et al*, 2002; Hughes *et al*, 2004; Altintas *et al*, 2007). This is likely to represent a significant clinical problem as the brain is a frequent site of relapse in melanoma. However, second-generation tyrosine kinase inhibitors such as dasatinib have shown some promise in Philadelphia chromosome-positive leukaemia patients with CNS involvement (Porkka *et al*, 2008). Furthermore, studies have looked at modulating the distribution of drugs such as imatinib by targeting P-glycoprotein and other drug protein transporters (Breedveld *et al*, 2005).

In conclusion, this report describes the clinical responses to KIT kinase inhibitors in melanoma patients harbouring diverse *KIT* mutations and supports the utility of selecting patients for therapy based on the identification of *KIT* mutations. This study did not test for *KIT* amplifications, which may also represent a melanoma subset sensitive to kinase-directed therapy. The demonstrated responses support the clinical testing of KIT inhibitors in the adjuvant setting in which potentially sensitive mutations in *KIT* have been identified, although the observation of frequent CNS relapse suggests that this may be a focus of ongoing research.

## Figures and Tables

**Table 1:** ‘At-risk’ population screening for *KIT* mutations

**Patient**	**KIT mutation**	**Melanoma subtype**	**Site**	**Primary/metastasis**
1	WT	Acral	Fifth finger	Met
2	WT	Acral	Thumb	Met
3	D820Y exon 17	Acral	First toe	Primary
4	WT	Acral	First toe	Met
5	WT	Acral	First toe	Met
6	WT	Acral	First toe	Met
7	WT	Acral	First toe	Met
8	WT	Acral	First toe	Primary
9	WT	Acral	First toenail	Met
10	WT	Acral	Sole of foot	Met
11	WT	Acral	Sole of foot	Primary
12	WT	Acral	Sole of foot	Met
13	WT	Acral	Sole of foot	Met
14	WT	Acral	Foot	Met
15	WT	Acral	Heel	Primary
16	WT	Acral	Heel	Met
17	7 codon dup exon 11	Mucosal	Anal	Met
18	D820Y exon 17	Mucosal	Anal	Met
19	V559A exon 11	Mucosal	Anal	Met
20	D816V exon 17	Mucosal	Rectum	Met
21	WT	Mucosal	Rectum	Met
22	WT	Mucosal	Rectum	Primary
23	WT	Mucosal	Nasal mucosa	Primary
24	WT	Mucosal	Ethmoid sinus	Primary
25	WT	Mucosal	Nasal septum	Met
26	WT	Mucosal	Vagina	Met
27	WT	Mucosal	Cervix	Primary
28	K642E exon 13	Mucosal	Vulva	Met
29	WT	Mucosal	Vulva	Met
30	WT	Mucosal	Vulva	Primary
31	WT	Mucosal	Vulva	Met
32	L576P exon 11	Mucosal	Labia	Met

Abbreviations: WT=wild type; Met=metastasis.

**Table 2:** Summary of treatment response according to *KIT* mutation status

**Patient**	**Mutation**	**KIT inhibitor**	**% RECIST**	**Best response**	**LDH × ULN baseline/best response**	**PET response**	**CNS relapse/progression**
1	21 bp dup exon 11	Imatinib	60	PR	1.1/0.8	Yes	Yes
2	K642E exon 13	Imatinib	35	PR	1.5/1.25	Yes	No
3	L576P exon 11	Imatinib	42	PD^a^	12.5/1.2	NA	Yes
4	D820Y exon 17	Sorafenib	27	SD	2.8/1.3	NA	Yes

Abbreviations: RECIST=response evaluation criteria in solid tumours; NA=not available, PR=partial response; SD=stable disease; PD=progressive disease; LDH=lactate dehydrogenase; ULN=upper limit of the normal/reference range; PET=positron emission tomography; CNS=central nervous system.

a New bladder lesion identified at first radiological assessment, despite dramatic clinical response and clear radiological response in pre-existing lesions.
